# A novel sensor-embedded holding device for monitoring upper extremity functions

**DOI:** 10.3389/fbioe.2022.976242

**Published:** 2022-11-03

**Authors:** Charlie Chen Ma, Pu-Chun Mo, Hsiu-Yun Hsu, Fong-Chin Su

**Affiliations:** ^1^ Department of Biomedical Engineering, National Cheng Kung University, Tainan, Taiwan; ^2^ Department of Physical Medicine Rehabilitation, National Cheng Kung University Hospital, Tainan, Taiwan; ^3^ Medical Device Innovation Center, National Cheng Kung University, Tainan, Taiwan

**Keywords:** biomechanics, rehabilitation, aging, upper extremities, robotic

## Abstract

There are several causes that can lead to functional weakness in the hands or upper extremities (UE), such as stroke, trauma, or aging. Therefore, evaluation and monitoring of UE rehabilitation have become essential. However, most traditional evaluation tools (TETs) and assessments require clinicians to assist or are limited to specific clinical settings. Several novel assessments might apply to wearable devices, yet those devices will still need clinicians or caretakers to help with further tests. Thus, a novel UE assessment device that is user-friendly and requires minimal assistance would be needed. The cylindrical grasp is one of the common UE movements performed in daily life. Therefore, a cylindrical sensor-embedded holding device (SEHD) for training and monitoring was developed for a usability test within this research. The SEHD has 14 force sensors with an array designed to fit holding positions and a six-axis inertial measurement unit (IMU) to monitor grip strength, hand dexterity, acceleration, and angular velocity. Six young adults, six healthy elderly participants, and three stroke survivors had participated in this study to see if the SEHD could be used as a reference to TETs. During result analyses, where the correlation coefficient analyses were applied, forearm rotation smoothness and the Purdue Pegboard Test (PPT) showed a moderate negative correlation [*r* (16) = −0.724, *p* < 0.01], and the finger independence showed a moderate negative correlation with the PPT [*r* (10) = −0.615, *p* < 0.05]. There was also a highly positive correlation between the maximum pressing task and Jamar dynamometer in maximum grip strength [*r* (16) = 0.821, *p* < 0.01]. These outcomes suggest that the SEHD with simple movements could be applied as a reference for users to monitor their UE ability.

## Introduction

The elderly population is rapidly increasing thanks to better medical support globally (Nation, 2019). Several studies have suggested that normal aging might alter physical, mental, and social health (Onder et al., 2002). Physical changes include decreased strength and mobility (Chodock et al., 2020). In particular, upper extremity (UE) functions and strategies may affect activities of daily living (ADL) due to physical changes in normal aging (Reissner et al., 2019). However, aging is not the only cause of UE dysfunction; these physical problems are also seen in patients who have suffered stroke (Shi et al., 2011), trauma (Dowrick et al., 2005; Crowe et al., 2020), Parkinson’s disease (Quinn et al., 2013), and other UE musculoskeletal disorders (Huisstede et al., 2006) such as carpal tunnel syndrome (Yoshida et al., 2019). In addition, UE functions are important components of ADL capability and may impact the quality of life (QoL) (Kanti Majumdar, 2014). Therefore, assessing and monitoring UE function is critical for people with varying degrees of dysfunction.

Several clinical evaluation methods could be applied to evaluate one’s UE functions (Kim et al., 2016). However, most of them are not digitized and based on clinical observations. For example, the common assessment tool for UE are the Upper Extremity Functional Index (UEFI-20 and UEFI-15), which is a self-administered questionnaire consisting of activity lists. Users give a score to each activity based on its difficulty of completion. Healthy individuals can score 80 out of 80 in UEFI-20 or 100 out of 100 in UEFI-15 (13). Aside from the self-administered questionnaires, the Purdue Pegboard Test (PPT) is one of the most commonly used clinical evaluation tools for fine and gross motor dexterity and coordination. The participants perform tasks within the designed period and clinicians calculate their performances based on how many pieces (pegs) are placed in the desired locations (holes) during the tasks. It is a performance-based outcome measure (Sigirtmac and Oksuz, 2022). The normal range of the score can vary due to the different age groups and genders. For example, in the healthy male group aged 21 to 25 years, the average PPT is 15.44 ± 1.71 in the preferred hand task (Yeudall et al., 1986; Marvin, 2012). The PPT could evaluate one’s hand dexterity and UE gross movements. However, the outcome measure of the PPT presents the number of pieces that might not present one’s UE abilities on a different scale. It could be considered an overall evaluation tool instead of a segmented ability evaluation. Besides PPT, the Jamar dynamometer is a common clinical assessment instrument for upper extremities; it evaluates one’s grip-force. It is considered a gold standard tool with excellent validity and reliability in research and the clinic (Mathiowetz, 2002; Bohannon et al., 2006). However, similar to the PPT, the Jamar dynamometer requires at least one clinician to be on-site, which calls for additional human labor. Clinicians must be trained before collecting or grading subjects’ functions. An experienced clinician might grade differently from an inexperienced one when operating the same evaluation tool on the same patient. Therefore, some current clinical assessments might end up with more subjective results based on the clinician’s experience (Kim et al., 2013). Aside from that, several tests have ceiling effects, meaning healthy individuals might get total scores even though their hand functions may differ. Moreover, many people do not realize or deny functional changes in their UE with age, so they do not visit the clinic or hospital for further evaluations (Kim et al., 2016). Therefore, there is a need for an easily accessible device that can digitize the assessment results to provide a reference for clinicians, which will, in turn, also help the users.

Several wearable smart gloves have been invented to help people with UE functional impairments perform training or rehabilitation (Wang et al., 2014; Shin et al., 2016). However, such wearable smart gloves might not be suitable for all users. Researchers have developed data gloves embedded with different sensors, such as inertia measurement unit (IMU) sensor-embedded gloves (Hsiao et al., 2015; Lin et al., 2017; Lin et al., 2018), data gloves with force sensors (Tarchanidis and Lygouras, 2003; Hsiao et al., 2015), data gloves with flexible sensors (Tognetti et al., 2006), and gloves with flexible optic fiber bending transducers (Fujiwara et al., 2014). However, studies have shown that the elderly resist the adoption of assistive technology for reasons such as (A) the devices not being easy to set up; (B) the devices being so expensive that they are afraid of breaking or losing them; (C) or the caretakers not bothering to help them put these on (Yusif et al., 2016; Golant, 2017). Aside from that, although some of the data gloves use force sensors, many of the force sensors in the gloves do not provide actual force acquisition due to erroneous placement of the sensors. Most wearable devices use pseudo-augmented reality environments (screen projections) or virtual reality environments to integrate with the hardware. However, the lack of tactile feedback of the actual object might lead to inappropriate force application during training or rehabilitation. Although smart gloves are considered a potential developing technology and many companies and research groups are devoted to these in this field, most wearable smart gloves are still not widely used (Caeiro-Rodríguez et al., 2021).

Grabbing is one of the earliest reflexes in human development (Halverson, 1937), while gripping and grasping are intuitive movements in daily living (Yang et al., 2015). The three holding-related verbs might be confusing. The movements of gripping, grasping, and grabbing are all similar since they use the hands to hold and apply appropriate force to keep and move the object. These movements are also different in that they require different reaction times to make contact with the object, e.g., grabbing represents a quicker action than gripping. In other words, the grasping movement tends to be more planned, while gripping is more intuitive. In this article, we will use “grasping” to present the planned holding movement. Generally, there are two major types of grasping: power grasp and precision grasp. Power grasp, which means force application, is the key element in movements such as the large wrap, cylindrical grasp, and power sphere grasp. Precision grasp, which means the stability of the object or trajectory of the following movement, is more important than the force application of the movement, such as the writing tripod and precision sphere. In these two types of grasping, several subtypes are categorized by the involvement of the digits, grasping shapes, and purposes (Yang et al., 2015; Cai et al., 2016).

Grasping is a complex movement that involves motor planning and the proper use of force application. Many muscles and the nervous system are involved in grasping (Castiello, 2005; Koester et al., 2016; Chen et al., 2022). Different people can employ different strategies when performing the same task and respond differently to different outcomes. Studies have shown that motor learning and motor control improve with more sensory inputs during movements (Chiu et al., 2009; Cano-De-La-Cuerda et al., 2015; Sani and Shanechi, 2021). The complexity of grasping can enhance brain activation by observing the environment (occipital cortex), understanding and planning movements (prefrontal cortex), and performing responsive movements (premotor cortex, primary motor cortex, and complementary motor cortex).

Overall, sensory feedback collected during grasping may help improve force application and motor performance. Therefore, the idea of innovating a grasping device for evaluation, monitoring, and rehabilitation was initiated and developed.

The purposes of this study are to (A) develop the novel sensor-embedded holding device (SEHD) for functional evaluation of grip-force and movements and (B) apply the SEHD to collect useful data that could further be used as references for clinicians to monitor UE functions.

The hypothesis of this study is that the SEHD could offer data, which after being processed, could provide results that can be considered as references to traditional evaluation tools (TETs) for users and clinicians to monitor the conditions of the users’ UE functions.

## Design of upper extremities evaluation device

### Design of hardware and its purposes

According to previous studies related to handgrips, holding devices could provide higher degrees of freedom to perform various movements, such as lifting, rotation, and horizontal position modification (Harwin and Barrow, 2013; Turella and Lingnau, 2014; Yang et al., 2015). In addition, studies related to grip force during movements suggest that data recorded from surface pressure or force sensors appear to facilitate further analysis to understand patterns of hand movement and hand function (Hsu et al., 2021a; Chen et al., 2022). Therefore, the design of the SEHD includes an inertial motor unit (IMU) that could provide speed and angular profiles. Based on this, we also encouraged the development of SEHD to collect sensory feedback.

Overall, the SEHD was designed and developed around several goals: 1) the SEHD should be capable of acquiring digitized data from different hand movements and UE movements; 2) it should be used and accepted by different users, such as the elderly, people with hand/UE disabilities, those who had survived a stroke, and children with developmental delays; 3) it should be affordable for the public and provide valuable analysis for monitoring, assessment, and even auxiliary training in the future. Therefore, designing and developing a proper SEHD appearance with useful functional details was very important and challenging.

### Development of hardware and data collection

The cylindrical grasp is considered one of the common UE movements in daily living (Vergara et al., 2014). The cylindrical design of the SHED comes from a widely accepted everyday item—the soda can (diameter: 65 mm and height: 140 mm), which is a widely accepted daily object. Relative research supports the idea that the cylindrical shape for grasping was easy to use and could offer a nice grip feeling (Harih and Dolšak, 2014). A diameter of 65 mm was decided in accordance with the study on grip strength (Domalain et al., 2008) and the average hand length (Guerra et al., 2014) such that the SEHD could be wrapped comfortably by the participants. The initial design focused on the functionality of the device. The commercial circuit board and sensors were adapted for the SEHD.

We used 3D printing to build the shell, which consisted of two materials: polylactic acid (PLA) and thermoplastic elastomer (TPE). PLA was used for the hard shell of the case, while TPE was used for the elastic, flexible cover of the force sensors. Red and white were used for different materials because people tend to stick their palms to the colored areas intuitively. The weight of the object was 200 g, and the weight of the device linked to the IC board was 500 g.

**FIGURE 1:**
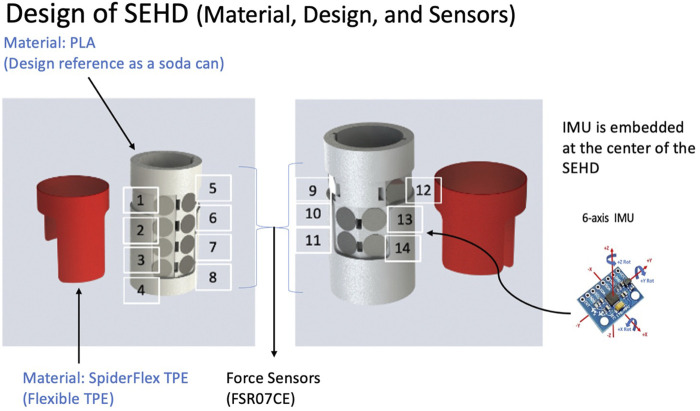
Design of the sensor-embedded holding device. Fourteen pressure sensors (FSR07CE, Ohmite) and the placement of the pressure sensors. Six-axis inertial measurement unit placed at the center of device. Inner part of structure made of PLA and outer part of the holding piece made of TPE material, and a 3D printer made both parts.

**FIGURE 2:**
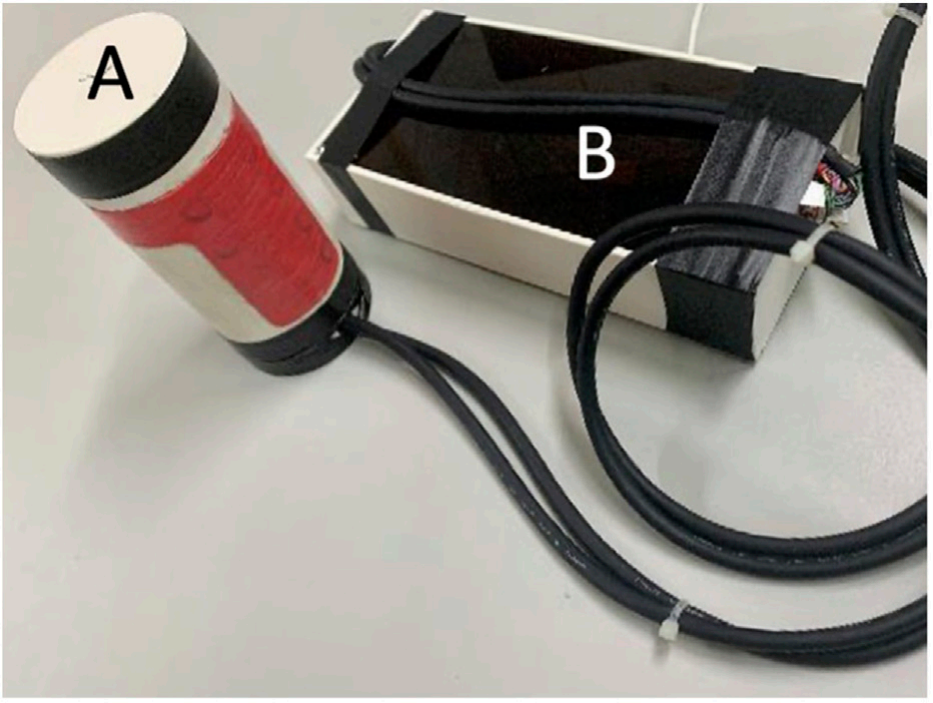
Actual product of SEHD. **(A)** Actual product of SEHD. **(B)** Process board of SEHD.

**FIGURE 3:**
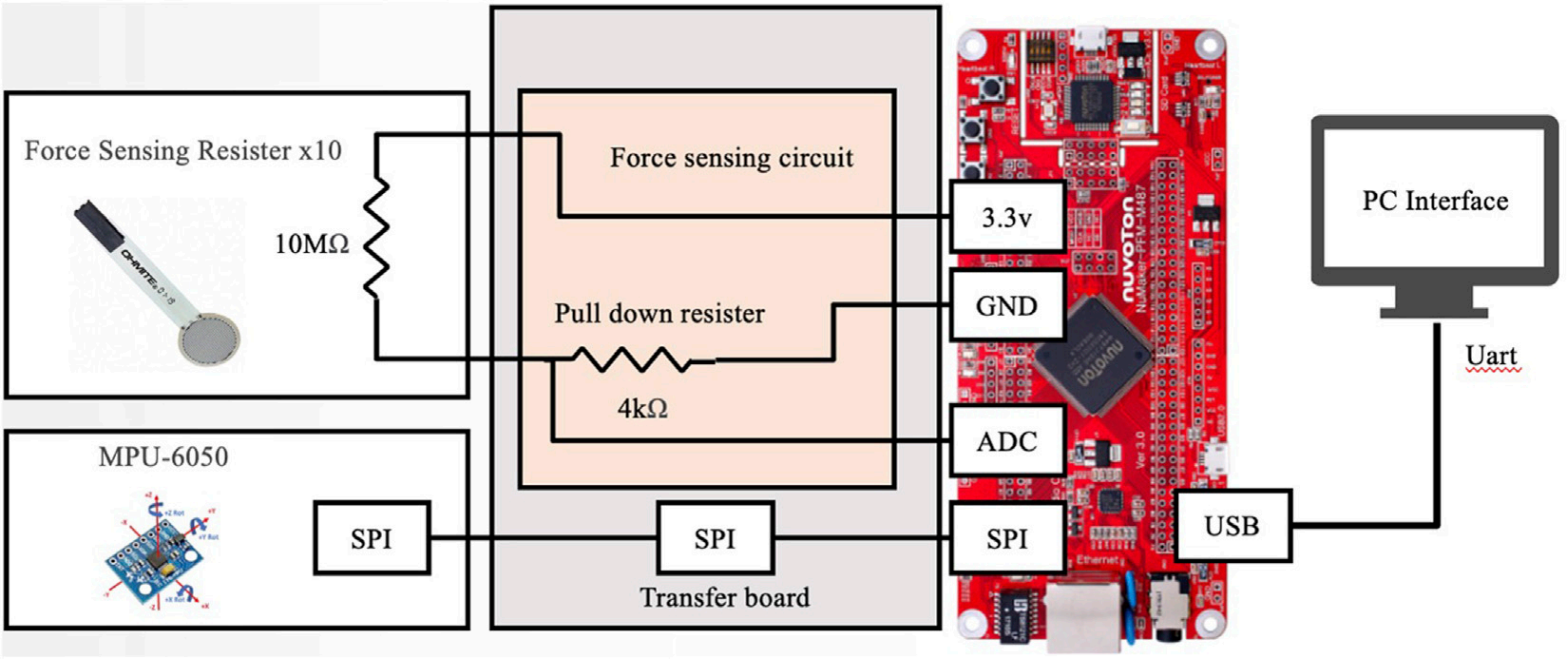
Circuit layout of SEHD: 14 force sensors linked to processing board (NuMaker-PFM-M487, Nuvoton, Taiwan) and IMU (MPU-6050, GY-521, InvenSense Inc., Taiwan) linked to SPI transfer board to transfer data to processing board. Processing board transfers data into digital output using micro-USB to the computer.

A six-axis inertia measurement unit (IMU) and 14 force sensors were embedded in the SEHD (Figure 1). The IMU has been widely applied in many devices to evaluate or monitor different movement tasks (Hsu et al., 2021b; Nikkhoo et al., 2021). The embedded IMU was an MPU-6050 sensor (GY-521) from InvenSense, which has been applied in many studies related to motion control, wearable device innovation, and movement sensation inputs (Ngui and Lee, 2019; Karnik et al., 2020; Ares et al., 2022). The validity and reliability of this model of IMU have been demonstrated in many of the previous studies that have been accepted for clinical applications. The IMU was placed at the center of the device so as to measure movement parameters, and the force sensors were placed under the TPE cover. Wires linked the processing board with the device. The output of the processing board provides data transfer and power to a PC or laptop *via* micro USB (Figure 2, Figure 3).

### Development of software

**FIGURE 4:**
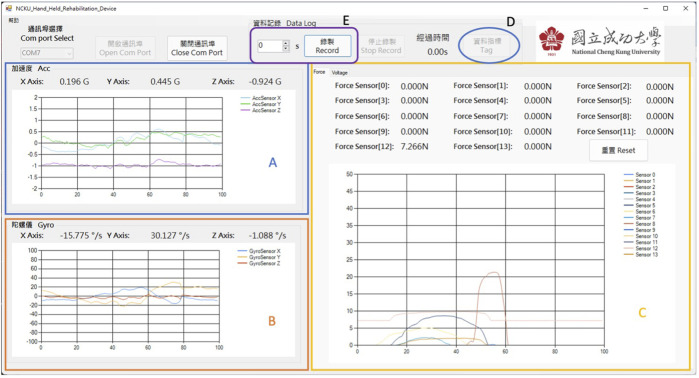
Software of SEHD: offers real-time monitoring during experiments. **(A)** Acceleration data; **(B)** angular velocity; **(C)** force sensor data: the reset button is for calibration; **(D)** “tag” button used as cues for different movement segments; **(E)** “record” button to preset the duration of each movement.

During initial design, the core functionality of the SHED software was to monitor device connectivity, monitor IMU and force sensor data in real time, and acquire data from the device. As shown in Figure 4, the interface design is dominated by simplicity and focuses on providing appropriate information. In particular, the data acquisition frequency was 100 Hz, and the IMU and force sensors were calibrated. The data were processed through the software, and the unit of each axis of the accelerometers is “G,” while that of each axis of the gyroscope is “degree/second.”

In addition, we had added some function buttons to improve user experience for the researchers. The a “calibration” button was used to zero the pressure from the elastic cover to the force sensors. A “Tag” button was designed for researchers to provide voice prompts to the participants during the experiment while also providing a “Tag” time point in the final output data. The adjustable duration could offer a fixed time for the researcher to conduct the tasks. After pressing the “record” button, the participants started performing the designed task. After the task was completed according to the preset duration, a pop-up window prompted that the data had been recorded and the storage path of the data.

## Methods

### Participants

Three groups of participants were recruited for this study: healthy young adults, healthy elderly, and stroke survivors. Fifteen participants (six healthy young adults, six healthy elderly, and three stroke survivors) were recruited based on the calculation of the sample size.

The calculation of the sample size was based on the textbook “Designing Clinical Research” by Hulley (2007). The ɑ of the two-tailed test was set as 0.05, *β* was set as 0.20, and *r* = 0.6 or 0.7. The results of the sample size would be N = 19 when *r* = 0.6 and N = 13 when *r* = 0.7. Therefore, 15 participants were recruited.

The inclusion criteria for healthy young adults were

1) aged between 20 and 30 years;
2) having no neurological or musculoskeletal disorders;
3) having had no UE surgery within past 6 months.

The inclusion criteria for the healthy elderly were

1) aged above 65 years;
2) having no neurological or musculoskeletal disorders;
3) having had no UE surgery within past 6 months;
4) being able to understand and follow instructions.

The inclusion criteria for the participants who had survived a stroke in the chronic stage were

1) survived more than 6 months after a stroke;
2) experienced only one stroke;
3) having no other neurological or musculoskeletal disorders;
4) having had no UE surgery within past 6 months;
5) capable of performing the Purdue Pegboard Test;
6) having no other cognitive impairment and being able to follow verbal instructions.

All participants were fully informed about the study and had signed the IRB-approved consent form of the National Cheng Kung University, Taiwan.

### Procedure of study

The procedure was separated into two parts: 1) clinical traditional evaluation methods and 2) novel SEHD simple tasks. The traditional evaluation tools included 1) the Jamar dynamometer to evaluate maximum grip strength and 2) the Purdue Pegboard Test (PPT) to assess fine movement and hand dexterity.

The background information on each participant was collected before the traditional evaluation. This background information included name, gender, date of birth, and upper extremity dominance side. Three groups of participants were included in this study to increase the diversity of the users.

The Purdue Pegboard Test (PPT) was conducted according to the guidelines of the clinical tutorial. The PPT is a common clinical evaluation tool for UE and hand movement. The participants were asked to start the PPT assessment from the dominant side, the nondominant side, and with both hands and to do the assembly task. The first three parts of the PPT made the participants place pegs in the holes from the top to bottom. The researcher would offer verbal cues like “Start” and “Time is up” to the participants. Thirty seconds were given to the participants, and the researcher would count how many pegs had been placed within the 30-s period. The assembly task required the participants to assemble a set of pieces, which included a peg, washers, and a nut. The participants were asked to assemble the set by alternating their hands and placing the set from the top to bottom. The given time was 1 minute, and the researcher would count how many pieces had been placed and assembled correctly within this period. Three trials were given for each task. The participants could ask for rest during the PPT.

After the PPT assessments had been completed, the participants were asked to use the Jamar dynamometer to assess their grip-force, which is considered a clinical gold standard test for assessing grip-force. Three trials were collected for each participant with breaks of 1 minute between the trials. The purpose of the 1-min break was to avoid fatigue that might affect the results of the trials. The participants were asked to sit comfortably on a chair and hold the Jamar dynamometer in an upright position. When the researcher says “grasp,” the participants had to grasp as hard as they could until the researcher says “rest.”

Based on the task activities using the Jamar dynamometer and PPT that have been described above, we divided each activity and its characteristics into three task groups: forearm rotation, maximum grip strength, and sequential press (see Table 1).

**TABLE 1:** Clarification of designed tasks and clinical meanings.

Task	What do I want to see	Related body parts	Parameters	Clinical implications
Forearm rotation	Rotation smoothness/rotation stability	Wrist	Angular speed profile	Related to daily functional activities
Maximum grip strength	Grip strength	Fingers	Force	Grip strength
Sequential press	Finger independence/finger coordination/holding position	Fingers	Force	Functional ability related to grip/fine movements

All movement data were collected while the participants were sitting and holding the SEHD with their dominant hand. We verbally instructed and offered demo movement before collecting the data. There was no verbal instruction given during the experiments, but we provided sound cues for participants to understand and perform the related movements. The reasons for using sound cues (beep) during the experiment are that 1) sound cues are shorter and quicker for the participants to respond to, especially since the movement duration is relatively short; 2) it avoids additional cognitive burden, since sound cues are more intuitive than verbal cues that need more processing; and 3) it avoids any opportunity for researchers’ mistakes when offering the wrong verbal cues. The participants were asked to place their fingers at the desired places. Five trials were conducted for each task, and the average was calculated in post-processing as the performance ability of the participants.

In the first movement task—the forearm rotation task—the participants were asked to sit comfortably in front of a desk such that their forearms could be placed on the desk at the resting position. The participants were asked to hold the SEHD restfully with their fingers placed on the red TPE area before the experiment started. They were asked to respond by raising the SEHD to an appropriate height when they heard the first sound cue (beep). When the second sound cue occurred, they had to respond by performing pronation, mimicking the action of pouring water from a cup parallel to the table. When the third sound cue occurred, they had to respond by performing supination back to the original position and wait for the last cue to complete the trial. After the trial, the participants were asked to place the SEHD back on the desk and wait for the next trial or the next task. The collection duration of each trial was 15 s. The participants could ask for a resting period between the trials.

In the second task—the maximum grip strength task—the participants were asked to sit restfully in front of the desk. The participants were asked to hold the SEHD restfully with their fingers placed on the red TPE area before the experiment started. The participants were asked to apply maximum grip when they heard the first beep and relax when they heard the second beep. Because the force application of the grip is a fast and quick movement that usually lasts less than 3 s (Kamimura and Ikuta, 2001), the duration of this task was designed to be 10 s and the duration of the compressions was to be less than 4 s to avoid additional fatigue. The participants were asked to take at least 1 minute of rest between each trial.

In the third task—the sequential pressing task—the participants were asked to perform sequential independent finger compressions from the index finger to little finger and from the little finger to index finger. A complete “sequential press trial” consisted of recording eight finger presses. A metronome offering 75 beats-per-minute (bpm) (0.8 s/cue) indicated that when the participants heard the first beep, they could start performing the task. They were asked to follow 75 bpm for each digit pressing and releasing (pressing for 0.8 s and releasing for 0.8 s). Once the full round of the sequential pressing trial (the index finger to little finger and the little finger to index finger) was completed, the participants were asked to hold their positions until the last sound cue occurred. The duration of each trial was 20 s.

The participants who had survived a stroke were only asked to collect three trials for the first two tasks, while the other participants were required to collect five trials for all three tasks. Fewer trials were collected due to the lower physical abilities of the participants who had suffered a stroke. The tasks were performed on both sides (affected and less affected) in order to gain a better understanding of the SEHD performance and TETs. The stroke survivors were not asked to perform the third task due to a lack of control of each digit and the poor understanding of the task.

### Data collection and statistical analysis

The designed software collected task performance data in real time, and the default collection frequency was 100 Hz. After the collection process, the system automatically transferred the data to a text file for further processing.

The results of the first task (forearm rotation) were processed in order to identify the “movement phase” and “holding phase.” The “movement phase” is how we calculate the movement’s smoothness. First, we had to identify the duration of the movement such that we could further calculate movement smoothness. There are several methods to calculate the moving phase from the data of the IMU, and we used one of the common ways to calculate the movement by finding the peak of acceleration and then 5% of the acceleration as the initiation of the action. Similarly, to find the termination of the movement. The time between the initiation and termination of the movement was considered the duration of the movement. Movement smoothness was calculated during the movement phase so as to understand the quality of the movement. Log dimensionless jerk (LDLJ) (Eq. (1)) was applied to represent movement smoothness (Gulde and Hermsdörfer, 2018; Melendez-Calderon et al., 2021). The results of the parameters were analyzed using the Pearson correlation coefficient with the Purdue Pegboard Test in order to understand the correlation between the SEHD and traditional evaluation tools.

Log dimensionless jerk (angular velocity)

$$\lambda_{L}^{\omega }\left(\omega \right)≜-\ln\left(\frac{{\left(t_{2}-t_{1}\right)}^{3}}{\omega_{peak}^{2}}\int_{t_{1}}^{t_{2}}{\left\|\frac{d^{2}}{{dt}^{2}}\omega \left(t\right)\right\|}_{2}^{2}dt\right),$$

$$\omega_{peak}≜\max_{t\in \left[t_{1},t_{2}\right]}{\left\|\omega \left(t\right)\right\|}_{2}.$$ (1)

ω represents angular velocity, where ω(t) represents the angular velocity of a movement in the time domain, and t_1_ and t_2_ are the start and stop times of the movement. The parameters that were applied in the following analysis were the duration of the movements (pronation and supination) and the LDLJ of the movements. Pearson’s correlation coefficient was applied to compare the correlations between the parameters and results of the PPT.

Pearson’s correlation coefficient was applied to compare the maximum grip strength of the device and results of the Jamar dynamometer.

The process of the sequential press was to calculate the percentage of total digit force application to the designed digit force application during the digit-pressing interval (Eq. 2). The results of the parameters were compared with the Purdue Pegboard Test so as to understand the correlation between the novel device and traditional tools by using Pearson’s correlation coefficient.

Finger independence

$$Finger\;Independence=\frac{F_{disired\;digit}}{F_{total\;digits}}\times 100\%$$ (2)

All data were processed using MATLAB software (MathWorks, Natick, Massachusetts, United States ) and analyzed by SPSS (SPSS Inc., Chicago, Illinois, United States ).

## Results

### Participants’ demographic information and traditional evaluation tools

There were three groups that participated in the study. The demographic information is listed in Table 2. The selection of the groups and the number of groups have been explained in the abovementioned Methods section. All the participants were right-side dominant.

**TABLE 2:** Demographic information of participants.

Group	Number	Age (mean ± SD)	Gender (male:female)	Dominant side of UE (right:left)
Healthy young adults	6	21.13 ± 0.46	4:2	6:0
Healthy elderly	6	64.18 ± 4.70	3:3	6:0
Stroke survivors	3	74.9 ± 4.16	0:3	3:0

The results from the traditional evaluation tools showed that stroke survivors performed the worst among the three groups across all assessments. Healthy young adults performed slightly better than healthy older adults on all outcomes assessed (Table 3).

**TABLE 3:** Results of assessment conducted by traditional evaluation tools.

		Purdue Pegboard Test (dominant)		Purdue Pegboard Test (both)		Purdue Pegboard Test (assembly)		Jamar dynamometer (dominant)	
Group	Number	Mean	SD	Mean	SD	Mean	SD	Mean	SD
Healthy young adults	6	16.61	1.10	13.17	1.41	42.67	8.80	30.67	14.53
Healthy elderly	6	15.33	0.63	12.17	0.91	36.61	4.46	23.25	2.87
Stroke survivors	3	11.5	1.32	9.67	0.29	23.17	2.47	14.25	3.88

All the stroke survivors had ischemic strokes on the left side of the brain. All of them had had a stroke during the past 6 months (time since stroke = 9.3 ± 3.5 years). The average Fugl-Meyer motor score for the affected side was 28.3, while the average Fugl-Meyer motor score for the less affected side was 36.3.

### Forearm rotation (pronation and supination)

There were two movement phases in this task: wrist pronation and supination. After applying the Pearson’s correlation coefficient analysis, there was a medium negative correlation between the duration of supination with the dominant-side hand task in the PPT [*r* (Yeudall et al., 1986) = −0.493, *p* < 0.05], both-hands task in the PPT [*r* (Yeudall et al., 1986) = −0.469, *p* < 0.05], and the assembly task in the PPT [*r* (Yeudall et al., 1986) = −0.488, *p* < 0.05] (Table 4). There was a moderate-to-high negative correlation between the movement smoothness of pronation and the dominant-side hand task in the PPT [*r* (Yeudall et al., 1986) = −0.724, *p* < 0.01], medium-to-negative correlation between the smoothness of pronation with both-hands task in the PPT [*r* (Yeudall et al., 1986) = −0.479, *p* < 0.05], and the assembly task in the PPT [*r* (Yeudall et al., 1986) = −0.535, *p* < 0.05].

**TABLE 4:** Correlation between the Purdue Pegboard Test and SEHD data during forearm rotation movement task.

Correlations				
	Duration of supination	LDLJ of pronation	LDLJ of supination	
Purdue (dominant)	Correlation coefficient	−0.493^a^	−0.724 **	−0.498^a^
	Sig. (2-tailed)	0.038	0.001	0.035
	N	18	18	18
Purdue (both)	Correlation coefficient	−0.469^a^	−0.479^a^	−0.421
	Sig. (2-tailed)	0.050	0.044	0.082
	N	18	18	18
Purdue (assembly)	Correlation coefficient	−0.488^a^	−0.535^a^	−0.423
	Sig. (2-tailed)	0.040	0.022	0.082
	N	18	18	18

^a^ Correlation is significant at the 0.05 level (2-tailed).

### Maximum grip

The purpose of this movement was to understand whether to use affordable sensors to measure maximum gripping force. After the Pearson’s correlation coefficient analysis, there was a highly positive correlation between the maximum grip force and Jamar dynamometer [*r* (Yeudall et al., 1986) = 0.821, *p* < 0.01] (Table 5).

**TABLE 5:** Correlation between Jamar dynamometer and data from SEHD during maximum grip task.

Correlation		
	Maximum grip task	
Jamar dynamometer	Correlation coefficient	0.821^a^
	Sig. (2-tailed)	0.000
	N	18

^a^ Correlation is significant at the 0.01 level (2-tailed).

### Sequential press

According to the designed task, the finger pressuring force could represent the independence of each digit if the participant could complete the task in sequence. After the Pearson’s correlation coefficient analysis of the finger independence, there was a moderate-to-negative correlation between the middle-finger independence and the right-hand subtask of the PPT [*r* (Yoshida et al., 2019) = −0.615, *p* < 0.05], medium-to-negative correlation between the middle-finger independence, and both-hand task of the PPT [*r* (Yoshida et al., 2019) = −0.565, *p* < 0.05] (Table 6).

**TABLE 6:** Correlation between Purdue Pegboard Test and data from SEHD during sequential pressing task.

Correlations			
	Finger independence (index finger)	Finger independence (middle finger)	
Purdue (dominant)	Correlation coefficient	−0.448	−0.615^a^
	Sig. (2-tailed)	0.082	0.011
	N	12	12
Purdue (both)	Correlation coefficient	−0.072	−0.565^a^
	Sig. (2-tailed)	0.790	0.023
	N	12	12

^a^ Correlation is significant at the 0.05 level (2-tailed).

## Discussion

### Purdue Pegboard Test and device-based processed data

The Purdue Pegboard Test is a standardized and widely used evaluation tool of hand dexterity and upper extremity functions. The movement of the Purdue Pegboard Test could be segmented into the following phases: short lifting, the transition of the objects, picking up the object, and placing the object at the desired spot. If the designed tasks are based on these segmented movements, using the SEHD could offer proper correlations with the Purdue Pegboard Test and could be considered a proper reference for clinicians to monitor one’s upper extremity functions and/or hand dexterity.

The results showed a medium-to-moderate negative correlation between supination duration and moving forward the tasks. The duration of a movement is one of the indicators of movement ability; the shorter the movement duration, the better the movement ability. According to the calculation of movement smoothness (Balasubramanian et al., 2011), a larger LDLJ meant worse performance of movement smoothness. The results of the studies showed a moderate-to-negative correlation between the pronation and supination of the wrist movement and the traditional Purdue Pegboard Tests of the dominant side, both-hand side, and assembly.

However, the results do not appear to offer a high correlation, which might be due to the following reasons: (A) although there were three groups of participants, most of them were healthy individuals despite differences in age. In addition, the movements were designed according to activities of daily living, which means that these tasks were easy to perform for individuals without any upper extremity disability; (B) the Purdue Pegboard Test results were rendered, which meant that the results of the PPT could not offer details on each segment’s upper extremity abilities. Therefore, each individual might apply different strategies during the test, and the same individual might even apply different strategies in each object-picking process; (C) there were ceiling effects and learning effects in healthy individuals conducting the Purdue Pegboard Test (Tseng et al., 2017); (D) movement smoothness (LDLJ) was calculated using the period of the movement unit and the acceleration change during the movement unit. Therefore, LDLJ and the Purdue Pegboard Test might not correlate highly with each other.

Overall, correlation is one of the important indicators for measuring the quality of mobility movements. Although the results of the SEHD may not correlate well with the Purdue Pegboard Test, it can still be considered a useful monitoring device for assessing hand and upper extremity abilities.

Another task related to the Purdue Pegboard Test is using the SEHD for the sequential pressing tasks. Since finger independence is one of the indicators of hand dexterity, the correlation between calculated finger independence and the Purdue Pegboard Test might offer relative references in understanding one’s dexterity.

The results show that the middle finger offered a better correlation coefficient with the Purdue Pegboard Test. This may be due to the different roles of the index finger and middle finger. The index finger is the primary guide when performing precise movements, while the middle finger is more supportive and adjustive in the modification of the movement. Finger independence is presented as percentage, with a higher percentage representing poorer finger independence. Therefore, the negative correlations between the Purdue Pegboard Test and percentage of middle finger independence are consistent with our observations.

### Jamar dynamometer and device-based processed data

The correlation results between the Jamar dynamometer and the data obtained from the SEHD during the maximum grip task are the most important results that can be derived from this study. This suggests that the application of affordable force sensors on this device can provide a reference for monitoring one’s power gripping ability and that this device can be used for the rehabilitation and monitoring of people with hand disabilities. Furthermore, the result also offers the reasonable belief that applying affordable force sensors to the device could provide references in monitoring one’s power gripping ability. Therefore, it is possible to use this device in the rehabilitation and monitoring of people with hand disabilities.

### Novelty of sensor-embedded holding device

The idea of creating a holding device was to provide a possible solution for the clinical issues in evaluating the hand and UE functions. These issues included 1) undigitized data (Marvin, 2012; Chesworth et al., 2014; Kim et al., 2016), 2) subjective data collection (Marvin, 2012), 3) removal of additional human labor (Bohannon et al., 2006; Sigirtmac and Oksuz, 2022), 4) complicated newer technologies for most of the users (Yusif et al., 2016), and 5) newer devices that were not focused on the force (Tognetti et al., 2006; Lin et al., 2018).

The development of the SEHD was to solve the above problems. The data collected by the SEHD were digitized and could be applied as useful clinical relevant parameters after processing. The shape, appearance, and weight of the SEHD were designed to provide a more intuitive user experience, remove technical barriers for users, and shorten the learning time for users to operate the device. Force sensors that were used in the SEHD could collect appropriate data, and the integration of the IMU and force sensors could provide more parameters related to UE abilities that most of the existing devices could not achieve.

### Limitations and future research

The sample size of the study was small, yet the primary purpose of this study was to introduce a novel monitoring device and demonstrate its operability. Studies with larger sample sizes will be considered in future planning. Another limitation would be the choice of the traditional evaluation tools, as such tools might not be able to correlate comprehensively with the SEHD. The concept of the movement tasks for the SEHD was to mimic the partial movement segments of the Purdue Pegboard Test. The PPT is a much complicated movement test, and the score of the PPT is a rendered result that includes UE gross movement, hand dexterity, hand–eye coordination, and control of placement. Therefore, applying other UE movement–related evaluation tools or designing other complex movements for the SEHD might be required.

There are several studies that reveal that grasp is highly related to cognitive rehabilitation (Morganti, 2004; van Vliet et al., 2013) that could be applied to other groups for rehabilitation or training purposes, such as for stroke survivors in order to improve their external focus (Durham et al., 2014) and for people with mild cognitive impairments (Cosgrove, 2016).

Therefore, future research may focus on the following directions: (A) continue to develop the SEHD as a rehabilitation intervention for different populations, such as for people who have suffered stroke, people with mild cognitive impairment, people with carpal tunnel syndrome, and examining the outcomes after an intervention; (B) continue to improve the SEHD’s capabilities, such as changing the wired SEHD to a wireless one or changing the sensors to ones with better resolutions for researchers; (C) integrate the SEHD and gamification software to provide a better user experience in different monitoring and rehabilitation settings, such as in clinics, in institutes, or when performing home activities (Lin et al., 2021). With the programmed interface, users can operate the SEHD alone with minimum assistance, which could benefit the users and caregivers.

## Conclusion

The purpose of the study was to develop a novel digitized assessment device that could acquire digitized data and process functional parameters from the designed movements in order to present upper extremity functional abilities, such as grip-force and movement abilities. By comparing the processed data with that of other traditional clinical tools, the quantitative functional outcome measurements suggest that the SEHD system can be used as a monitoring tool for users in need. The integrated hardware and software in the SEHD offer proper usability and feasibility for people who need UE rehabilitation, allowing them to understand their upper extremity ability at home or in hospitals.

Based on this research, SEHD processed data could be considered as references for users in understanding their UE functional abilities.

## Data Availability

The original contributions presented in the study are included in the article/Supplementary Material; further inquiries can be directed to the corresponding author.
