# Supplementary material for: A novel sensor-embedded holding device for monitoring upper extremity functions
Source: Front Bioeng Biotechnol. 2022 Nov 3;10:976242. doi: 10.3389/fbioe.2022.976242 (PMC9670142; doi:10.3389/fbioe.2022.976242)
Supplement: Supplementary file 1 [file Presentation1.PPTX]

## Slide 1
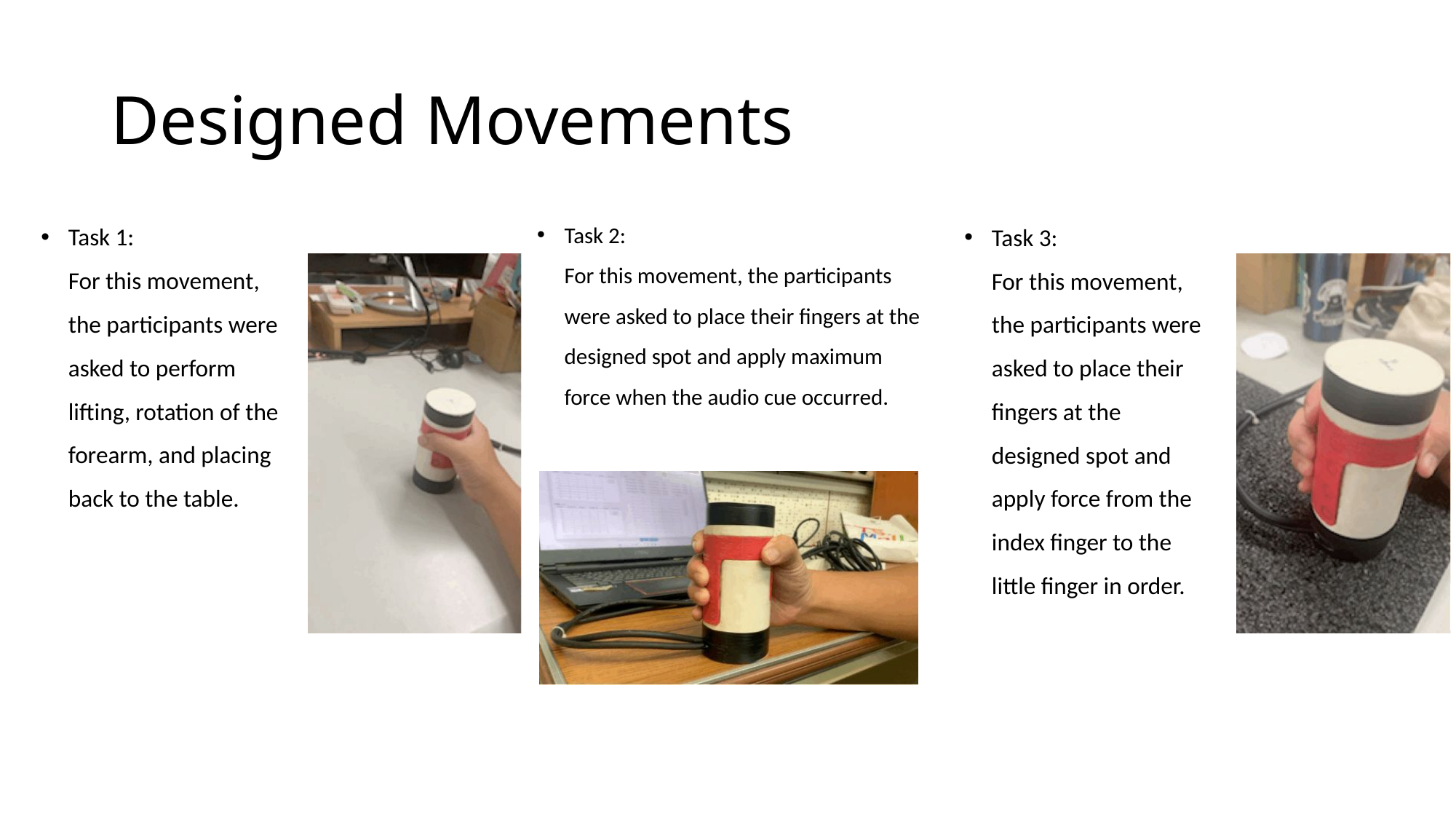

# Designed Movements
Task 1:
For this movement, the participants were asked to perform lifting, rotation of the forearm, and placing back to the table.
Task 2:
For this movement, the participants were asked to place their fingers at the designed spot and apply maximum force when the audio cue occurred.
Task 3:
For this movement, the participants were asked to place their fingers at the designed spot and apply force from the index finger to the little finger in order.
