# Supplementary material for: A novel sensor-embedded holding device for monitoring upper extremity functions
Source: Front Bioeng Biotechnol. 2022 Nov 3;10:976242. doi: 10.3389/fbioe.2022.976242 (PMC9670142; doi:10.3389/fbioe.2022.976242)
Supplement: Supplementary file 2 [file Table2.DOCX]

Naming system:

In “all processed data”:

- “t1”: task 1: Wrist rotation task
- “t2”: task 2: Maximum grip task
- “t3”: task 3: Sequential pressing task
- Purdue_D: dominant side task
- Purdue_B: both hands task
- Purdue_As: assembly task
- “dm1”: duration of movement unit 1
- “dm2”: duration of movement unit 2
- “ldljm1”: Log Dimensionless Jerk of movement unit 1 (movement smoothness)
- “ldljm2”: Log Dimensionless Jerk of movement unit 2
- “t3_in”: independence index of the index finger during task 3
- “t3_mid”: independence index of the middle finger during task 3

In “basicInfo”: (basic information about the participants)

- “DomSide”: dominant side of the subject
- “PPT_D”: Purdue pegboard test of a dominant side task
- “PPT_ND”: Purdue pegboard test of a non-dominant side task
- “PPT_B”: Purdue pegboard test of both hands task
- “PPT_As”: Purdue pegboard test of the assembly task
- “Jamar”: the results of Jamar dynamometer

In “task1_wrist_rotation”:

- “d_m1”: duration of movement unit 1
- “d_h1”: duration of holding phase 1
- “PPV_m1”: peak per velocity in movement unit 1
- “LDLJ_m1”: Log Dimensionless Jerk of movement unit 1

In “task2_max”:

- “Side”: “1” represents dominant side of the subjects
- “Maxforce”: maximum force measured by the force sensor of the thumb as the reference during the task 2
- “Jamar”: Jamar dynamometer

In “task3_fingerIndependence”:

- “t3_index”: independence index of the index finger during task 3
- “t3_middle”: independence index of the middle finger during task 3
